# Embracing a new phase: Ribosome binding promotes phasiRNA biogenesis

**DOI:** 10.1093/plcell/koae298

**Published:** 2024-11-13

**Authors:** Michael Busche

**Affiliations:** Assistant Features Editor, The Plant Cell, American Society of Plant Biologists; Laboratory of Genetics, University of Wisconsin, Madison, WI 53706, USA

Phased secondary small interfering RNAs (phasiRNAs) are short sequences of RNA that play important roles in development, immunity, and abiotic stress tolerance in plants (Liu et al. 2020; Yang et al. 2021; Li et al. 2022). The precise timing of their expression and accumulation is critical during maize anther development, as some mutants lacking or mis-expressing phasiRNAs are male-sterile. phasiRNAs are generated from so-called *PHAS* loci that are transcribed into phasiRNA precursors. These precursors are recognized by specific microRNAs, which guide Argonaute to cleave them into either 21- or 24-nucleotide (nt) molecules. While phasiRNA precursors have been thought to be primarily noncoding RNAs (i.e. not encoding functional peptides), they contain small open reading frames (sORFs) that potentially could be translated. In fact, it was recently shown that some phasiRNA precursors are bound by ribosomes (Yang et al. 2021). A new study in *The Plant Cell* by **Yingjia Han and colleagues (Han et al. 2024)** investigated this phenomenon across the stages of maize anther development, focusing on the production of 24-nt phasiRNAs and the importance of ribosome binding in this process.

Han and colleagues used a combination of ribosome profiling (Ribo-seq), small RNA sequencing, and RNA sequencing (RNA-seq) to examine both the possible ribosome binding and transcription of 24-nt phasiRNA precursors in developing anthers. Ribo-seq is an approach in which only ribosome-bound RNAs are sequenced, providing a snapshot of the transcripts actively bound by ribosomes at a given moment. This analysis, in tandem with traditional RNA-seq, showed that the total 24-nt phasiRNA precursors (Fig., magenta bars) and ribosome-associated 24-nt phasiRNA precursors (Fig., purple bars) both peaked at the 1.5-mm stage. The small RNA sequencing results showed that 24-nt phasiRNAs peaked in abundance when anthers were 2.0 mm in length. The correlation between ribosome binding and phasiRNA precursor peaking time during anther development raised questions about whether ribosome binding, typically associated with translation, might play a role in producing mature phasiRNA molecules.

**Figure.**
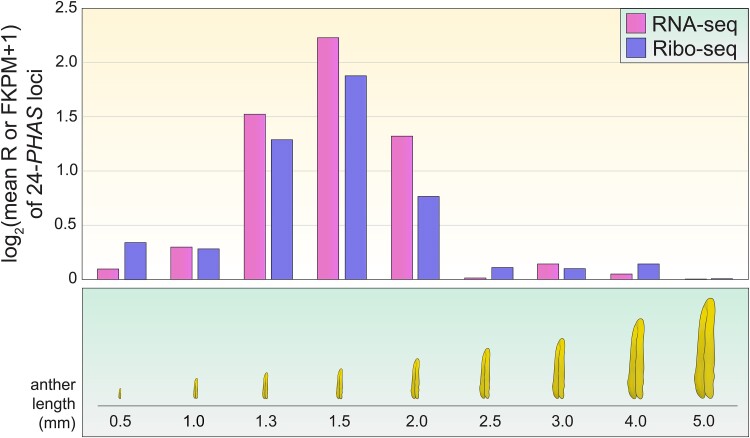
The 24-nt phasiRNAs precursor abundance and binding by ribosomes peak at the same stage in anther development. Han et al. performed RNA-Seq (magenta bars) and Ribo-seq (purple bars) on anthers throughout development, from 0.5 mm long to maturation at 5.0 mm long. They found that 24-nt phasiRNA precursors increased during early anther development, peaking at the 1.5-mm stage and decreasing in abundance thereafter. The number of 24-nt phasiRNA precursor fragments bound by ribosomes followed this same pattern. Furthermore, the abundance of 24-nt phasiRNAs themselves (not shown) peaked at the 1.5- to 2-mm stage as well, closely following the peaks of 24-nt phasiRNAs precursors and ribosome binding. Figure designed by M. Busche; data from Han et al. (2024) Table S3.

To test if ribosome binding is important for mature phasiRNA biogenesis, the team used CRISPR to generate mutations in a 24*-PHAS* locus that would only impair ribosome recognition while preserving other aspects of phasiRNA biogenesis, including microRNA binding. This targeted deletion indeed eliminated ribosome binding but also caused a ∼50% reduction in mature 24-nt phasiRNAs from this locus. Importantly, the phasiRNA precursor levels remained unchanged, supporting the idea that ribosome binding itself contributes to phasiRNA processing. Moreover, disruption of the sORFs in the ribosome-binding region, such that a new sORF was generated, did not impair ribosome binding to the phasiRNA precursors or affect phasiRNA production. This result demonstrates that recognition of an sORF by a ribosome per se, rather than the potential peptides translated from these sORFs, enhance the accumulation of 24-nt phasiRNAs.

It is still unclear how exactly recognition by ribosomes is involved in phasiRNA processing. The researchers suggest that the ribosome may provide physical support for the next steps in processing or help other translation-associated factors to stabilize or accelerate phasiRNA processing. The absence of translated peptides from these sORFs suggests a third hypothesis: the ribosome stalling and failing to proceed with translation may trigger the subsequent steps of phasiRNA processing.

## Data Availability

Data is available at https://doi.org/10.1093/plcell/koae289.
